# Cardiac Autonomic Modulation in Domestic Cats with Obstructive Lower Urinary Tract Disease

**DOI:** 10.3390/ani14233479

**Published:** 2024-12-02

**Authors:** Mary Marlene Tarazona Molina, Priscylla Tatiana Chalfun Guimarães-Okamoto, Amanda Sarita Cruz-Aleixo, Miriam Harumi Tsunemi, Jaqueline Valença Corrêa, Jessica Cardia de Melo, Luiz Henrique de Araújo Machado, Maria Lucia Gomes Lourenço

**Affiliations:** 1 Department of Veterinary Clinics, School of Veterinary Medicine and Animal Science, São Paulo State University (UNESP), Botucatu 18618970, Brazil; mary.tarazona@unesp.br (M.M.T.M.); tatiana.okamoto@unesp.br (P.T.C.G.-O.); amanda.cruz21@hotmail.com (A.S.C.-A.); jaqueline.v.correa@unesp.br (J.V.C.); jessica.cardia@unesp.br (J.C.d.M.); henrique.machado@unesp.br (L.H.d.A.M.); 2 Institute of Biosciences, São Paulo State University (UNESP), Botucatu 18618689, Brazil; m.tsunemi@unesp.br

**Keywords:** autonomic nervous system, obstruction, cardiology, potassium

## Abstract

Obstructive feline lower urinary tract disease (OFLUTD) is the most common complication of the urinary system, as metabolic and electrolyte changes can alter the functioning of the autonomic nervous system (ANS). The objective of this study was to describe the indices of heart rate variability (HRV) and their correlations with the observed alterations in Systolic Blood Pressure (SBP) and electrocardiographic, biochemical, and haemogasometric indices in cats diagnosed with OFLUTD. Sixty-five male cats up to 10 years of age were divided into two groups. The control group (CG) was composed of 25 healthy cats, and the obstructed group (OG) consisted of 40 cats with OFLUTD. The OG was evaluated by electrocardiography and blood pressure at four different time points. In the CG, electrocardiographic evaluation and SBP measurement were performed. A comparison of the HRV between the CG and OG (M0) revealed differences in the SDNN (standard deviation of all normal RR-NN) parameters (sympathetic and parasympathetic tone) and in the rMSSD (parasympathetic tone); there was a difference in the SBP, which was greater in the CG. There were higher rates in the CG. The HF and HR were greater in OG. The HRV serves as a preventive tool and predicts the severity of OFLUTD in patients due to an imbalance in the ANS.

## Simple Summary

OFLUTD may present imbalances in the autonomic nervous system and hypothalamic-pituitary-adrenal axis, activating the sympathetic nervous system (SNS) and releasing catecholamines. We suggest that describing HRV indices in the short term in cats diagnosed with OFLUTD and investigating the influence of autonomic modulation on the pathophysiology of the disease can provide beneficial clinical management, which can be used as a predictor of ANS imbalance and increase patient survival. Obstructed patients had lower temperatures, evidencing the influence of uremic toxins on thermoregulation; increased total proteins, segmented neutrophils, lymphocytes, decreased platelets and electrolyte changes such as hypercalcaemia, hypocalcaemia, low bicarbonate levels (HCO_3_); and decreased pH, in addition to exhibiting high levels of urea and creatinine. Arterial chemoreceptors are involved in the autonomic balance of the heart and are sensitive to changes in arterial gas pressure and pH. There was a decrease in HRV in animals with OFLUTD and an improvement due to treatment. The evaluation of HRV parameters such as SDNN and the rMSSD could serve as preventive tools and aid in determining patient prognosis due to the imbalance of the ANS, as these parameters can provide prompt assistance and reduce the risk of death.

## 1. Introduction

One of the most common diseases in feline medicine is feline lower urinary tract disease (FLUTD), which can be obstructive or nonobstructive. Obstructive disease is one of the most common emergencies in the urinary system [1], and different clinical signs, such as decreased glomerular filtration, altered regulation and resorption of sodium and water, and reduced elimination of potassium, causing acidosis, uraemia, hyperkalaemia, and ionised hypocalcaemia, have been observed [2,3].

OFLUTD may present as imbalances in the autonomic nervous system and hypothalamic-pituitary-adrenal (HPA) axis, activating the sympathetic nervous system (SNS) and releasing catecholamines, mainly norepinephrine [4,5]. These changes can be caused by stimulation of bladder neurons, increased activation of the sympathetic nervous system, and increased catecholamines. In addition, the urine of these cats contains urotoxins that can stimulate other neurons [6].

Continued stimulation of the SNS may cause increased systemic blood pressure (SBP), cardiac arrhythmias, haemodynamic changes, or ischaemic processes that may lead to sudden death [7,8]. It is believed that sympathetic autonomic activation therefore correlates with the pathophysiology of the disease in cats.

One of the most commonly used parameters for assessing autonomic activity is heart rate variability (HRV), which can be calculated in the domain of time (Table 1 and frequency, with different indices used to evaluate autonomic activity. In addition to being a noninvasive method, the appropriate study of this parameter allows an adequate assessment of ANS (Autonomic Nervous System) activity because HRV is the result of the simpato-vagal balance sheet of the ANS [9].

The evaluation of HRV can be performed by the Holter method, frequency audiometer cardiac and manual (ECG), which provides pathophysiological information on the state and functioning of the ANS in patients with OFLUTD because the imbalance can be related to urinary dysfunctions and electrolyte alterations in these animals [10,11].

Thus, the objective of this study was to describe HRV indices in the short term in cats diagnosed with OFLUTD using a digital electrocardiograph for three minutes to investigate the influence of autonomic modulation on the pathophysiology of the disease, providing prompt assistance, benefiting clinical management and increasing patient survival, which can be used as a predictor of ANS imbalance, and increasing patient survival.

## 2. Materials and Methods

The study was carried out at the Faculty of Veterinary Medicine and Animal Science of UNESP—FMVZ—Botucatu, SP, after approval by the Ethics Committee on the Use of Animals (n° protocol ECAU 130/2022) and signing of the informed consent form by the guardians of the animals that participated in the study.

### 2.1. Animals and Study Site

We used 65 male cats with no defined breed or weight with a maximum age of 10 years; the cats were divided into two groups.

The obstructed group (OG) was composed of 40 male cats with OFLUTD (OG) treated at the Nephrology and Veterinary Urology Service of the Veterinary Hospital of FMVZ—Unesp, Botucatu Campus (SP), and as a control group (CG), 25 healthy male cats were used.

### 2.2. Inclusion and Exclusion Criteria of the Study

The OG included male cats with a diagnosis of OFLUTD lacking a defined time after obstruction and various numbers of episodes; the cause of the obstruction was urethral plugs (soft material containing minerals, cells, and mucus-like protein) and urinary stones; the cats included in the OG had been excluded from the study animals presenting another joint disease (osteoarthritis).

In the CG, only healthy animals were included (without changes in physical examination, electrocardiography, or SBP), and animals that were receiving medical treatment were excluded.

### 2.3. Anamnesis and Physical Examination

Weight (kg), body condition score (BCS), body temperature (°C), capillary filling time, heart rate (bpm), respiratory rate (RR) (mpm), hydration, mental status (alert, lethargic or stupor), femoral pulse, and SBP were evaluated. The animals were kept in a calm environment, in a specific room for felines, and were carefully handled to reduce stress.

### 2.4. OFLUTD Service Protocol

The animals treated presented symptoms for an average of 3 days. The animals were subjected to the treatment protocol of the Nephrology and Veterinary Urology Service of the Veterinary Hospital of FMVZ—Unesp, Campus of Botucatu (SP), which consisted of the following steps:

Evaluation of the animals;

ECG (before anaesthesia and sedation);

Sedation and blood collection for haematological, biochemical, and haemogasometric tests (blood samples were collected with the patient sedated);

Electrolytic replacement, if necessary, after haemogasometry;

Cistocentesis of allude (for the allude cystocentesis, ultrasound was not performed);

Patient stabilisation;

General and epidural anaesthesia;

Removal of urethral obstruction and clamping of the probe;

Bladder wash;

Treatment after clearance (from the outpatient clinic);

ECG;

Meloxicam SID (3 days, SC or IV);

Bladder washing (3 days);

Treatment after clearance (Home);

Amitriptyline (SID) (30 days);

Prazosina BID (7 days);

Environmental management and stress reduction (multimodal environmental modification—MEMO).

*Evaluation moments*

Four electrocardiographic evaluations and SBP measurements were performed in the OG group, with a time interval of 24 h between each of the moments:

- M0: First day of service;
- M1: 24 h after removal of the urethral obstruction;
- M2: 48 h after removal of the urethral obstruction;
- M3: 72 h after removal of the urethral obstruction.

Blood tests were performed only on the first day of care (M0) in the OG.

In the CG, parameters such as SBP, electrocardiogram, HRV, and physical examination were evaluated at one time point.

### 2.5. Laboratory Tests

The sample was taken prior to drug therapy. Laboratory tests (blood count, serum biochemistry, and blood gas analysis) were performed in the Clinical Laboratory of the Department of Veterinary Clinic of FMVZ—UNESP, Botucatu. Three blood samples were sent from the jugular vein for different analyses, serum biochemistry (clot activator tube for urea dosage [Ur—mg/dL], creatinine), haemogasometry [syringe with lithium heparin for evaluation [sodium (Na^+^—mmol/L), potassium (K^+^—mmol/L), bicarbonate (mmol/L), pH blood, excess base (mmol/L) chloride (Cl—mmol/L), ionised calcium (Ca—mmol/L)]], and blood count [tube with EDTA]).

### 2.6. Conventional Electrocardiographic Evaluation

Electrocardiographic evaluation was performed with the digital veterinary electrocardiograph TEB (Brazilian Electronic Technology, São Paulo-SP, Brazil) of simultaneous derivations (DI, DII, DIII, aVR, aVL, and aVF) at a speed of 25 mm/s and calibration for 1 mm corresponding to 0.1 mV. The cats were positioned on a steel table covered with a rubber-insulating surface to avoid possible interference. The examination was performed without sedation for three minutes. On examination, the patient’s heart rate was evaluated, and the amplitude and duration of the P, R, and T waves; duration of QRS complexes; and duration of the PR and QT segments were subsequently compared with those described for the feline species.

### 2.7. Evaluation of Systemic Blood Pressure

Noninvasive systemic blood pressure was assessed on the days of clinical examination, according to the protocol proposed by the Consensus Statements of the American College of Veterinary Internal Medicine (ACVIM 2018) [12], using the Doppler Medmega DV610 (MedMega, Franca-SP, Brazil) with a one-way sphygmomanometer. The cuff size was selected according to the diameter of the right or left chest limb so that the cuff width was between 30% and 40% of the limb circumference, the patient was accommodated in a comfortable position, the thoracic limb was slightly extended, and we took precautions to avoid stressing the animal. Ultrasound gel was used in the palmar region of the thoracic limb, specifically in the common digital artery, to measure systemic blood pressure [13]. Five measurements were performed, excluding the maximum and minimum values, and the arithmetic mean was obtained as the final value.

A reference interval between 120 and 140 mmHg was used. Intervals between 140 and 159 mmHg were considered low-risk prehypertensive agents, intervals between 160 and 179 mmHg were considered moderate-risk hypertension, intervals ≥180 mmHg were considered hypertension at high risk of target organ injury, and intervals smaller than 120 mmHg, along with symptoms such as weakness, syncope, and tachycardia, were considered hypotensive [12,14].

### 2.8. Heart Rate Variability (HRV)

The HRV was calculated in the time domain using the following indices: standard deviation of all normal RR or NN intervals (SDNN) (milliseconds), percentage of differences greater than 50 ms between normal RR intervals (PNN50), and the square root of the square mean of the adjacent normal RR interval differences in a time interval (rMSSD, expressed in milliseconds). The HRV indices in the frequency domain analysed using the fast Fourier transform (FFT) algorithm included high-frequency components (high frequency [HF]) ranging from 0.15 to 0.4 Hz, low-frequency components (low frequency [LF]) ranging from 0.04 to 0.15 Hz and LF/HF ratios [15,16]. The electrocardiographic examination was performed without sedation for three minutes to obtain VFC indices.

Kubios software (Biomedical Signal Analysis Group, Department of Applied Physics, University of Kuopio, Finland, ver 3.0.2) was used for analysis of the HRV, assessing HRV in the fields of time and frequency.

Table 1 shows the autonomic performance of the HRV indices in the domain of time and frequency.

**Table 1:** HRV indices in the domain of time and frequency and autonomic action.

HRV Index	Meaning	Autonomic Performance
SDNN	Standard deviation of all normal RR intervals recorded in a time interval (ms)	Sympathetic and parasympathetic activity; however, they do not allow us to distinguish when HRV changes are due to increased sympathetic tone or removal of vagal tone
rMSSD	Square root of the square mean of the differences between adjacent normal RR intervals in a time interval (ms)	Parasympathetic activity
pNN50	Percentage of adjacent NN intervals with duration difference greater than 50 ms	Parasympathetic activity
High Frequency (HF)	Variation between 0.15 to 0.4 Hz	Parasympathetic activity. It corresponds to respiratory modulation and is an indicator of the performance of the vagus nerve on the heart
Low Frequency (LF)	Variation between 0.04 and 0.15 Hz	Sympathetic activity Resulting from the joint action of the vagal and sympathetic components on the heart, with predominance of the sympathetic
LF/HF	LF/HF ratio	Simpato-vagal balance. Reflects the absolute and relative changes between the sympathetic and parasympathetic components of the ANS, characterising the patho-vagal swing over the heart

(Adapted from [17] Vanderlei et al., 2009).

### 2.9. Statistical Analysis

The Shapiro-Wilk test was used to assess the normality of the data, the *t* test was used for parametric data, and the Mann-Whitney test was used for nonparametric data. For multiple comparisons between the respective moments of the OG variables, the Friedmann test was used. All the results were considered significant when *p* < 0.05. The correlations between the various parameters (clinical, pressure, electrocardiographic, biochemical, and haemogasometric) were verified using Pearson’s test for parametric variables and Spearman’s test for nonparametric variables, with significant correlations at *p* < 0.05. In addition, receiver operating characteristic (ROC) curve analysis was used to evaluate the sensitivity and specificity of the concentrations of three parameters (potassium, urea, and creatinine).

## 3. Results

### 3.1. Clinical Parameters

A total of 65 male cats were evaluated and divided into two groups: the CG (*n* = 25), with an average weight of 5.0 ±1.0 kg and age of 4.2 ± 2.0 years; and the OG (*n* = 40), with an average weight of 4.76 ± 1.08 kg and age of 3.4 ± 2.0 years (Table 2). In the CG, clinical, electrocardiographic, and HRV parameters were evaluated at a single time because the cats were healthy.

A significant difference was observed between the CG and OG in terms of respiratory rate (RR), temperature, and SBP (*p* < 0.05), with the medians being greater in the CG than in the OG at M0; however, a greater RR than the reference values was observed in the CG, and a lower temperature than the reference values was observed in the OG. For SBP, values above the reference in the OG were also observed; prehypertension was observed at low risk in M0 (7.5%; *n* = 3/40), hypertension at moderate risk in M0 (5%; *n* = 2/40), high-risk hypertension in M0 (2.5%; *n* = 1/40) and hypertension in M0 (2.5%; *n* = 1/40).

Regarding the degree of hydration of the cats in the OG, 25% (*n* = 10/40) of the animals had normal hydration, but 75% (*n* = 30/40) had different degrees of dehydration [slight 45% (*n* = 18/40), moderate 27.5% (*n* = 11/40), severe 2.5% (*n* = 1/40)].

### 3.2. Haematological Parameters

According to the average and median haematological indices, most cats obstructed at the initial time point presented results within the reference parameters described for the feline species, except for a decrease in RDW (Red Cell Distribution Width) (20%; *n* = 8/40) and platelet count (50%; *n* = 20/40) and total protein (TP) elevation (65% *n* = 26/40), segmented neutrophil count (7.7% *n* = 31/40) and lymphocyte count (42%; *n* = 17/40). The values are arranged in Table 3. In the CG, laboratory parameters were not evaluated because the cats were healthy.

Most of the obstructed animals had increased urea (90%, *n* = 36), creatinine (85%, *n* = 34/40), potassium (85%, *n* = 34/40), and lactate (30%, *n* = 12/40) levels.

For the blood pH, OG presented an acidic pH (47.5%; *n* = 19/40) and a decrease in bicarbonate (HCO_3_) (52,5%; *n* = 21/40), chlorine (Cl^−^) (57.5% *n* = 23/40), ionised calcium (Ca2) (42.5% *n* = 17), and excess base (EB) (80% *n* = 32/40), as shown in Table 4. In the CG, laboratory parameters were not evaluated because the cats were healthy.

### 3.3. Electrocardiographic Parameters

ECGs were performed on the day of obstruction (M0, *n* = 40/40) and on the day of treatment (M1, *n* = 34/40; M2, *n* = 24/40; and M3, *n* = 15/40); predominantly, sinus rhythm was observed on Day M0 (60% *n* = 24/40), followed by a sinoventricular rhythm with an atrial parade (25% *n* = 10/40), a left anterior fascicular block (LAFB) (7.5% *n* = 3/40), a complete right branch lock (7.5% *n* = 3/40), and sinus tachycardia (>240 bpm) (7.5% *n* = 3/40). At M1, 88% (*n* = 30/34) of the patients exhibited sinus rhythm, 8.8% (*n* = 3/34) had sinus tachycardia, and 2.9% (*n* = 1/34) had LAFB. On M2, 91% (*n* = 22/24) of the patients exhibited sinus rhythm, and 8.3% (*n* = 2) exhibited sinus tachycardia. At the last time of analysis, M3 had a predominant sinus rhythm in 93% (*n* = 14/15) of the animals and in 6.6% (*n* = 1/15) of the animals from sinus tachycardia.

A significant difference was detected (*p* < 0.05) in the comparison of the different electrocardiographic parameters (axis, QT duration, and T-wave amplitude) among the OGs; the medians are within the values described for the feline species and are shown in Table 5.

There was a significant difference (*p* < 0.05) in the comparison of different electrocardiographic parameters between the CG and OG in M0 (QRS complex duration, QT interval duration, and T-wave amplitude), the median within the values described for the feline species, as shown in Table 6.

In M1, there was a significant difference between the CG and OG groups in the amplitude of the P-wave (mV) (0.02) and the PR interval (ms) (*p* = 0.00); in M2, there was a significant difference in P-wave duration (ms) and the PR interval (ms) (*p* = 0.00); and in M3, there was a difference in the PR interval (ms) (*p* = 0.00), with the median within the values described for the feline species.

### 3.4. Relationship Between Electrical Conduction Disorders and Laboratory Parameters in Cats in the OG (Potassium, Urea, and Creatinine)

**Figure 1:**
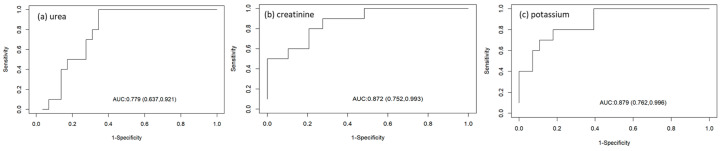
Receiver operating characteristic curves evaluating the accuracy of biochemical parameters ((**a**) urea, (**b**) creatinine, and (**c**) potassium) for determining sinoventricular rhythm with an atrial parade.

The accuracy of the biochemical parameters for determining the sinus rhythm with atrial arrest was also analysed (Table 7; Figure 1).

**Figure 2:**
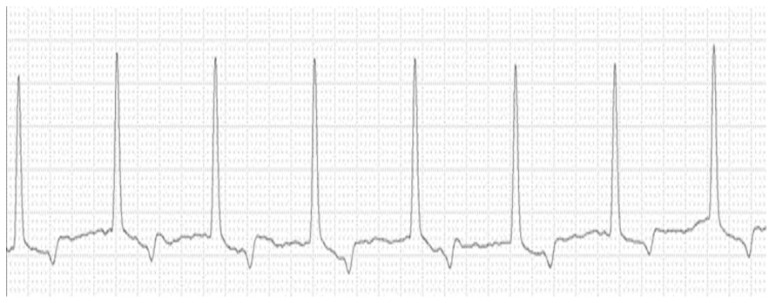
Electrocardiographic tracing of a cat with OFLUTD (front plane shunt II, 25 mm/s, 2 N sensitivity) showing sinoventricular rhythm with atrial paralysis (8.24 K^+^ mmol/L, 418 mg/dL urea and 16.02 mg/dL creatinine).

The creatinine and potassium concentrations presented an area under the curve (AUC) of 87%, but the parameter with the highest sensitivity (low rate of false-negatives) for determining the sinoventricular rhythm with atrial arrest was potassium (83%), with a cut-off point of 7.66 mmol/L. For specificity (a low rate of false-positives), the specificity of creatinine (80%) was greater than that of potassium (70%). Figure 2 shows the results of electrocardiographic tracing of a cat with a potassium concentration, urea concentration, and creatinine concentration higher than the cut-off points for each parameter for this cardiac electrical conduction disorder.

**Figure 3:**
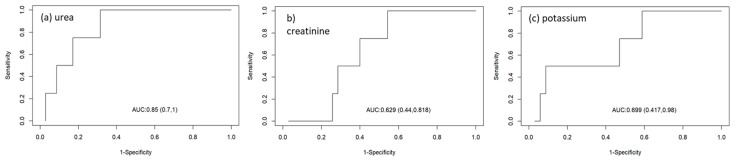
*Receiver operating characteristic curves* evaluating the accuracy of biochemical parameters ((**a**) urea, (**b**) creatinine, and (**c**) potassium) for determining right branch block.

The evaluation of the accuracy of biochemical parameters for the determination of right branch block urea concentration presented an area under the curve of 85%, in addition to greater sensitivity to right branch block (73%), with a cut-off point of 357 mg/dL and specificity of 60% (Table 8; Figure 3).

**Figure 4:**
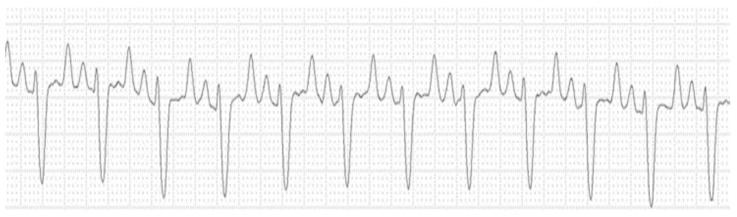
Electrocardiographic tracing of a cat with OFLUTD (front plane shunt II, 25 mm/s, 2 N sensitivity) with sinus rhythm and a right branch block (6.68 K^+^ mmol/L, urea 517 mg/dL, and creatinine 14.66 mg/dL).

Figure 4 shows the electrocardiographic tracing of a cat with higher potassium, urea, and creatinine concentrations at the cut-off points for each parameter for this cardiac electrical conduction disorder.

**Figure 5:**
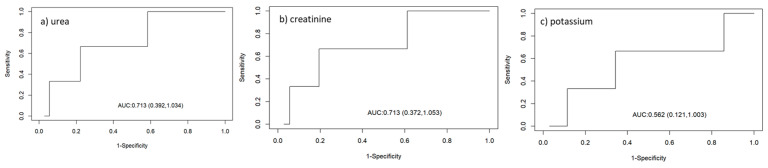
Receiver operating characteristic curves evaluating the accuracy of biochemical parameters ((**a**) urea, (**b**) creatinine, and (**c**) potassium) for the determination of left anterior fascicular block.

**Figure 6:**
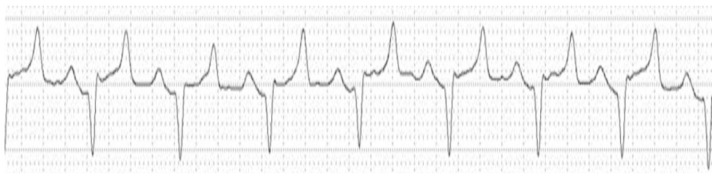
Electrocardiographic tracing of a cat with OFLUTD (front plane shunt II, 25 mm/s, 2 N sensitivity) presenting LAFB (8.36 K^+^ mmol/L, urea 355 mg/dL, and creatinine 11.99 mg/dL).

The accuracy of the biochemical parameters urea and creatinine in determining the left anterior fascicular block (LAFB) area under the curve was 71%, but the parameter with greater sensitivity to LAFB, creatinine (73%), had a cut-off point at a concentration of 4.91 mg/dL. The specificity for the three parameters was 0% (Table 9; Figure 5). Figure 6 shows the electrocardiographic tracing of a cat with higher potassium, urea, and creatinine concentrations at the cut-off points for each parameter for this cardiac electrical conduction disorder.

### 3.5. Evaluation of HRV and SBP

HRV indices were evaluated in 60% (n = 24/40) of OG animals in M0 because 40% (n = 16/40) of the cats presented different continuous arrhythmias during the three minutes of ECG due to electrolyte changes, preventing analysis at the present time. A significant difference (*p* < 0.05) was detected in the comparison of HRV at different moments and parameters; HRV was greater at m3, and the rMSSD was greater at M1 (Table 10).

There was a significant difference (*p* < 0.05) in the comparison of SBP at different time points in the OG; the median in M3 (145 mmHg), as described in Table 10, was greater.

A significant difference was detected in the HRV between the CG and OG for M0 (*p* <0.05) and in the SDNN (*p* < 0.00 ms) and rMSSD (*p* < 0.00) parameters, with the former being greater in the CG. The CG also exhibited greater mean NN, PNN50%, LF, and LF/HF values than did the CG. No OG or heart rate (HR) or HF were superior were used for CG (Table 11).

A comparison of the HRV indices between the CG and OG at all time points revealed that the mean NN, LF, and LF/HF indices were significantly greater in the CG; however, the rMSSD indices were significantly greater in the OG M1, SDNN, PNN50%, HF, and HR at M3.

**Figure 7:**
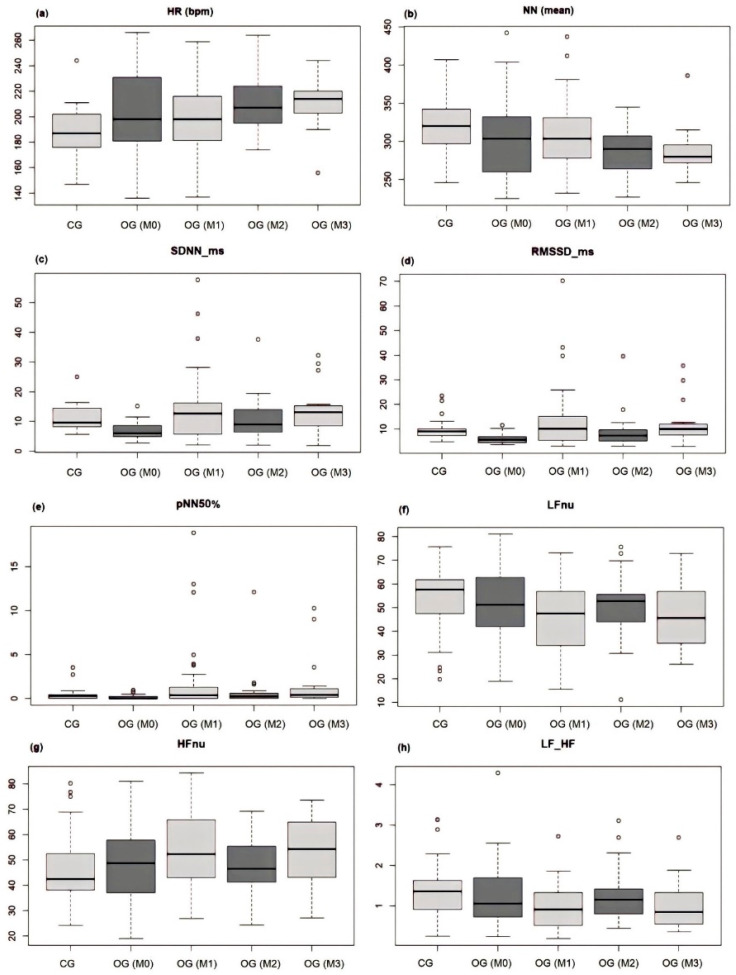
Comparison of HRV parameters in the CG versus OG at different times, with the values of parameters (**a**–**h**) on the vertical axis and the group and the moment [CG, OG (M0), OG (M1), OG (M2), and OG (M3) on the horizontal axis].

There was a significant difference (*p* < 0.05) in the comparison of HRV parameters between the CG and OG in M0 in the SDNN (*p* = 0.00) and the rMSSD (0.00); in M1 in the LF (*p* = 0.03), the HF (0.03), and the LF/HF (0.01); in M2 in the HR and NN (*p* = 0.00); and in M3 in the HR and NN (*p* = 0.00) (Figure 7).

**Figure 8:**
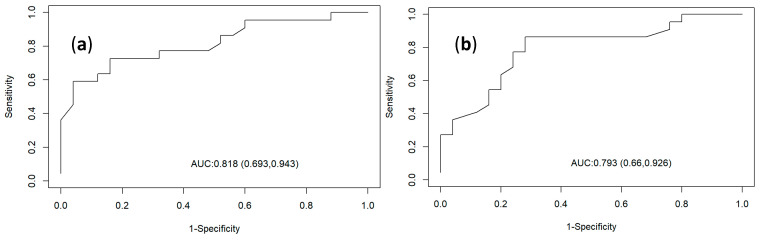
Accuracy of HRV parameters ((**a**) SDNN and (**b**) rMSSD) for the determination of OFLUTD, with the vertical axis indicating the sensitivity of the parameter and the horizontal axis indicating the specificity.

In the evaluation of the accuracy of the HRV parameters for the determination of OFLUTD, the SDNN index presented an AUC of 81%, with a sensitivity of 84%, specificity of 35%, and cut-off value of 8 ms. The rMSSD index was the second parameter with the highest percentage of area under the curve (Auroc) of 79%, with a sensitivity of 72%, specificity of 45%, and cut-off point of 7.8 ms (Figure 8).

When evaluating the correlations between HRV indices and potassium, urea, creatinine, and SBP in M0, significant positive correlations were observed only between SBP and LF indices (ꝑ < 0.05; *p* = 0.017) and between SBP and LF/HF indices (ꝑ < 0.05; *p* = 0.016); however, a negative correlation was observed for HFs (ꝑ < 0.05; *p* = 0.016).

## 4. Discussion

The aim of this study was to evaluate autonomic modulation in cats with OFLUTD (OG) at four different times after hospital admission and compare them with healthy cats (CG).

During the clinical examination, differences in the RR and temperature were observed, with the former being greater than the latter being. In the study by Dijkstra and Szatmári (2018) [18], in which the respiratory rate of 142 cats was evaluated, it was observed that healthy cats had a greater RR at the time of veterinary consultation before manipulation than at home, with an RR ranging from 32 to 135 (rpm) due to the highest excitation caused by the stress of finding themselves in a different place.

For temperature, the OG was below the reference temperature, which was associated with a change in the hypothalamic thermoregulatory centre caused by the uremic toxins of these animals. In addition, in one study, correlations were observed between the concentrations of creatinine, urea, phosphorus, magnesium, and pO2 and temperature, and a positive correlation was observed with pH [19] according to our study because OG is associated with an acidic pH.

In relation to haematological tests of the OG, the most relevant data on the blood count were increased total proteins, segmented neutrophils, and lymphocytes and decreased platelets and RDW. In agreement with the description by Torrente and Bosch (2011) [20], haemoconcentration (total proteins) due to dehydration and leukogram of stress (neutrophilia) [21] in addition to the inflammatory picture (lymphocytosis), decreased platelets, and related destruction and decreased bone marrow production caused by azotemia [22]. The SNS promotes the release of inflammatory markers related to pain and stress, which can increase catecholamine levels [11]. Inflammatory processes are usually associated with pain, are related to low HRV, and may serve as an indicator of stress [23,24].

As shown in the present study, the OG animals exhibited multiple changes in electrolyte levels, such as hypercalcaemia, hypocalcaemia, low bicarbonate levels (HCO_3_), and decreased pH, in addition to exhibiting high levels of urea and creatinine. Increased pH and lactate may be associated with dehydration and renal impairment, leading to decreased conditioning infusion [19]. Arterial chemoreceptors are involved in the autonomic balance of the heart and are sensitive to changes in arterial gas pressure and pH. Hypercalcaemia causes a reduction in the potential for cell rest, resulting in a more negative response (−90 mV) and a decreased excitability of cardiomyocytes [25], which may cause the development of cardiac arrhythmias. Due to the imbalance of the ANS, lower HRV rates were observed in patients with impaired renal function.

In the study by Alfonso et al. (2020) [26], it was observed that dogs with electrolyte disturbance-predictive parameters for arrhythmias and electrical conduction disorders had higher and, consequently, lower HRV; this finding is related to our study because it was observed that higher concentrations at the cut-off points of potassium, urea, and creatinine led to changes in cardiac electrical conduction, causing arrhythmias and cardiac electrical conduction disorders.

Patients with OG who presented sinus rhythm but still had urea, creatinine, and potassium concentrations above the reference values described for the feline species; however, with values lower than the cut-off points for each parameter, they presented lower HRV than did the CG. For the patients with higher concentrations at cut-off points (urea, creatinine, and potassium) who developed arrhythmias such as sinoventricular rhythm with atrial stop, right branch block, and LAFB, wherein the correlation with urea, creatinine, and potassium, it was observed that for the sinus rhythm with atrial arrest, the parameter with the highest determination was potassium; because, in addition to presenting an area under the curve (Auc) of 87%, this parameter presented a sensitivity of 83%, identifying true patients at risk of sinoventricular rhythm with an atrial parade and having a low rate of false-negatives; and because of its high specificity (70%), identifying patients who are not at risk of presenting a sinoventricular rhythm with an atrial parade and having a low rate of false-positives.

For the right branch block, the parameter with the greatest determination was urea, for which the area under the curve (AUC) was 85%, with a sensitivity of 73% (a low false-negative rate) and a specificity of 60%. For LAFB, the area under the curve was 71%, and the parameter with the highest sensitivity was creatinine (73%), but this parameter did not have good specificity (0%), as it was associated with a high rate of false-positives. The study of these predictive indices, in addition to assisting in the prognosis of HRV and in the prediction of arrhythmias, can provide insight into the concentrations of these parameters in places where it is difficult to carry out these tests, and further studies in feline species are needed.

Differences in the electrocardiographic parameters between the CG and OG at different moments and between the different moments of the OG were observed. In addition, several electrocardiographic changes in the OG were observed, such as an increase in the duration of the QT interval; in the QRS complex; in the P-wave and PR intervals; in the P-wave amplitude; in the sinoventricular rhythm; and in the right branch block, hyperkalaemia, hypocalcaemia, and acid-base imbalance, which may affect the excitability of the cell membrane [25], because high extracellular potassium concentrations decrease phase 0 (depolarisation) action potential, generating the lowest excitability of cardiomyocytes and decreasing the QT interval.

Furthermore, hypocalcaemia’s powerful changes caused by hyperkalaemia, in addition to extending phase 2 (potassium output), increased the QT interval. In this study, LAFB was observed in three animals, which is consistent with the findings of the study by Neri et al. (2016) [19], who also reported that LAFB was common in cats with feline hypertrophic cardiomyopathy [27]. Arrhythmia was associated with electrolyte and metabolic disorders in the patient, but after stabilisation, the felines exhibited sinus rhythm.

In relation to systemic blood pressure, the findings of that study agreed with those of Neri et al. (2016) [19], in which some of the animals presented different degrees of hypertension; moreover, there was a difference between the CG and OG at the initial time points M0 and in the OG at the different time points analysed. Stress is related to hypertension because the adrenaline secreted by the adrenal medulla stimulates the SNS by acting on presynaptic beta adrenergic receptors, causing norepinephrine release and consequently reducing baroreflex sensitivity, increasing SBP and HR but reducing HRV [28,29].

Hypertension was observed in the present study and was related to dehydration, decreased preload (volume), activation of compensatory mechanisms, stimulation of the baroreptors in the carotid sinus, increased SNS activation by the release of vasopressin and corticotropin (ACTH), increased release of catecholamines and cortisol, and increased cardiac frequency [30].

A comparison of the HRV indices between the CG and OG (M0) revealed that the OG (M0) presented higher hazard ratios (HRs) and, therefore, a shorter N-N interval; however, the CG presented all the indices in the time domain (SDNN, rMSSD, PNN50%), and the differences in the SDNN and rMSSD were greater than those in the OG, relating to the greater ANS and nervous parasympathetic system (PNS) [17]. The LF and LF/HF indices were greater in the CG than in the OG in M0 and M1, but the HF indices were greater in the OG (M0 and M1) than in the CG. In addition, in M1, differences were observed in all frequency domino indices. These indices do not directly represent the activity of the ANS, but they are believed to describe the balance of the sympathetic and parasympathetic systems, which can be altered by different disorders, reducing HRV [31].

Because HF is related to respiratory modulation, indicating the action of the vagus nerve in the heart, LF has a sympathetic predominance but results from the joint action of vagal and sympathetic action, as the LF/HF ratio reflects the simultaneous-to-vagal balance of the heart [17]. In addition, stressful and fearful conditions can alter the balance of the ANS without causing changes in HR but rather changes in HRV [32]. Regarding the comparison of HRV of CG versus OG M2 and M3, there was a difference in HR, being higher in the OG (M2 and M3) and NN higher in the CG, attributing the increase in HR to the recognition of the place because the HR of the OG was higher on the last day (M3) of care in the hospital.

A comparison of the HRV of the CG and all the OG timepoints revealed that the average NN medium, LF (sympathetic), and LF/HF (sympathovagal balance) indexes were greater in the CG and rMSSD (parasympathetic) cohorts, and the HR was greater in the OG M1, SDNN (ANS equilibrium), PNN50% (parasympathetic), and HF (parasympathetic) cohorts. Hyperkalaemia has depolarising effects on the heart, causing shortened action potentials and increasing the risk of arrhythmias, thereby reducing HRV indices. Furthermore, potassium channels in the myocardium are known to be sensitive to changes in endogenous factors and may change in number or function in response to structural factors. The increase in indices indicating greater parasympathetic or equilibrium activity of the ANS was related to the treatment (meloxicam, amitriptyline, and prazosin) given to these patients on Days M1, M2, and M3 because of the decrease in the symptoms that alter HRV, increasing parasympathetic activity. Meloxicam has anti-inflammatory effects on the peripheral nervous system (PNS), antipyretic effects on the central nervous system (CNS), and analgesic effects resulting from the action of meloxicam on the CNS and PNS up to 24 h after administration. In addition, meloxicam reduces stress in cats [33,34]. Changes in HRV have been observed in studies where low HRV is found in situations of pain and stress and increased SNS activity [35].

Amitriptyline also has sympathiatric properties, anticholinergics, antihistanics, analgesics, and anti-inflammatory drugs, providing greater activation of the PNS, reducing clinical signs, and increasing HRV in OG [36,37,38] due to the reduction in uptake of norepinephrine and serotonin, activating negative feedback mechanisms, leading to stress or anxiety reduction [39,40] having a half-life of eight to fifty hours, reaching its maximum concentration between two to 12 h, after oral administration [38] effect on M1, causing changes in HRV.

Prazosin is used to reduce ureteral muscle spasms through interactions with the SNS because it is an antagonist of the adrenergic receptor α1 that decreases the stiffness of the smooth musculature of the urethra [41,42,43] and reduces noradrenergic stimulation [44]. Usually, the maximum concentration is reached in three hours, with a half-life of 10 h, [10] an effect from the moment of administration, leading to changes in HRV during the evaluation.

In the assessment of the accuracy of the HRV parameters for the determination of OFLUTD incidence, the parameter with the greatest correlation was the SDNN because, in addition to having an area under the curve (AUC) of 81%, this parameter had a high sensitivity (84%), identifying true patients at risk of presenting OFLUTD and having a low rate of false-negatives but a low specificity (35%) (false-positives). rMSSD was the parameter with the second highest percentage of area under the curve (AUC) (79%), with a sensitivity of 72% and specificity (false-positives) higher than that of SDNN (45%).

In the review carried out by [45] He et al. (2022), possible biomarkers for the onset of the disease were described, but there is still a lack of evidence of sensitivity and specificity, in addition to the economic aspect and the speed of the test; therefore, the test could be HRV because its indices could help prevent OFLUTD, as cats with lower HRV indices at the described cut-off points (SDNN, 8 ms; rMSSD, 7.8 ms) are more likely to develop OFLUTD. The other HRV parameters presented an area under the curve (AUC) lower than 70%.

Among the correlations of HRV parameters with potassium, urea, creatinine, and SBP in M0, we observed differences in the domino indices of frequency; a positive correlation between SBP and LF and between the LF/HF ratio; and a negative correlation between SBP and HF. These findings showed that as SBP increased, the index of sympathetic predominance lf also increased because, in hypertension, greater activation of the SNS was observed, with reduced HRV [46,47], while the opposite was observed for HF because it has a parasympathetic predominance, decreasing SBP. The LF/HF ratio reflects the absolute and relative changes in the NHS and PNS in the heart [17].

In the present study, we observed that medications, electrolytic stabilisation, and a reduction in urea and creatinine levels in patients modified HRV indices, with an increase in some indices in the OG at different times compared to those in the CG. Like in Alfonso et al. (2020) [26]’s study in dogs with chronic renal insufficiency, in which they had electrolytic and biochemical disorders, HRV indices increased after patient stabilisation.

One of the limitations of the study was the physical restraint of the animals, mainly from the CG, and the high concentrations of norepinephrine and epinephrine probably reached after the first minute of stress [45,48], stimulating the SNS and changing the HRV. In addition, the wide range of BUN used in this group may also have been a limiting factor, as HRV decreases as age progresses [16], making it difficult to compare a healthy group with a group of sick cats. The evaluation time also limited the study because the treatment of sick cats interferes with HRV, suggesting that future studies with longer evaluation times in healthy and sick cats (after home treatment) would help provide a greater understanding of the disease.

## 5. Conclusions

The short-term evaluation of HRV showed that male cats diagnosed with OFLUTD had a greater sympathetic predominance than healthy animals with a lower HRV. It was possible to evaluate autonomic modulation in cats with healthy animals with OFLUTD through an electrocardiogram. In addition to observing arrhythmias due to electrolyte disturbances in these animals, there was also the possibility of assessing the ANS in a short time, as there was a decrease in HRV in animals with OFLUTD and an improvement due to treatment.

The evaluation of HRV parameters such as SDNN and the rMSSD could serve as preventive tools and aid in determining patient prognosis due to the imbalance of the ANS, as these parameters can provide prompt assistance and reduce the risk of death. However, further studies are needed to better elucidate ANS modulation in obstructive lower urinary tract disease in cats.

## Figures and Tables

**Table 2:** Means, standard deviations, and medians of clinical parameters evaluated in cats in the OG (n = 40) at the initial time point (M0).

Physical Examination/Parameters	CG (n = 25) Mean ± SD (Median)	OG (n = 40) Mean ± SD (Median)	Interval Reference	*p*
Weight (kg)	5.0 ± 1.0 (5.0)	4.76 ± 1.08 (4.6)	-	0.0260 ^a^
Age (years)	4.2 ± 2.0 (4.0)	3.4 ± 2.0 (3.0)	-	0.098 ^b^
RR (rpm)	57.84 ± 13.34 (52.0)	35.93 ± 21.18 (28)	20–40	0.000 ^b^*
T (°C)	38.46 ± 0.58 (38.5)	37.13 ± 1.12 (37.1)	37.8–39.2	0.000 ^b^*
SBP (mmHg)	132.12 ± 18.83 (130.0)	120.82 ± 26.847 (120)	<140	0.03 ^b^*

Mean ± SD: standard deviation (median). Significant *p* < 0.05; * *t* test *t* (^a^) and Mann-Whitney test (^b^).

**Table 3:** Haematological parameters of OG cats at M0. Mean, SD: standard deviation, median, maximum (Max) value, and minimum (Min).

Parameters	Mean ± SD (Median)	Max	Min	Interval Reference
RDW %	12.05 ± 6.62 (15)	17.70	0	14.00–19.00
Platelets/μL	235.00 ± 175.5 (282)	555.50	0	300–800
TP Plasma g/dL	8.42 ± 0.92 (8.4)	10.60	6.40	6.00–8.00
Segmented/μL	115.40 ± 131.20 (51.65)	515.00	6.20	2.500–12.500
Lymphocytes/μL	7.74 ± 7.91 (6)	27.90	0.20	1.500–7.000

Biochemical and haemogasometric parameters.

**Table 4:** Mean, standard deviation, and median biochemical and haemogasometric parameters in M0 cats in the OG.

Parameters	Mean ± SD (Median)	Max	Min	Interval Reference
Urea (mg/dL)	321.01 ± 233.48 (277.00)	1284.00	29.60	42.80–64.20
Creatinine (mg/dL)	10.39 ± 6.39 (10.90)	23.74	1.05	0.80–1.80
pH	7.21 ± 0.11 (7.19)	7.43	6.98	7.24–7.40
HCO_3_ (mmol/L)	14.94 ± 3.65 (14.80)	22.70	7.50	17.00–21.00
BE (mmol/L)	−11.31 ± 4.89 (−11.40)	−2.10	−20.10	−2.00–+3.00
Cl^−^ (mmol/L)	107.12 ± 6.54 (107.00)	120.00	96.00	111.00–125.00
K (mmol/L)	6.44 ± 1.86 (6.64)	9.91	3.15	3.90–5.30
Ca_2_ (mmol/L)	1.09 ± 0.194 (1.09)	1.40	0.76	1.10–1.40
Lactate (mmol/L)	2.14 ± 1.26 (1.70)	5.70	0.60	0.50–2.00

Mean, SD: standard deviation, median, maximum (Max) value, and minimum (Min).

**Table 5:** Comparison of ECG parameters among cats in the OG at different times in order of the mean, standard deviation, and median.

ECG/OG	M0 (n = 40) Mean ± SD (Median)	M1 (n = 34) Mean ± SD (Median)	M2 (n = 24) Mean ± SD (Median)	M3 (n = 15) Mean ± SD (Median)	*p*
Eixo (°)	25.1 ± 76.94 (54)	77.20 ± 41.48 (82)	73.2 ± 60.5 (86.5)	78.6 ± 44.70 (90)	0.03 ^b^*
P (ms)	40 ± 10 (40)	30 ± 0.00 (30)	30 ± 0.00 (30)	40 ± 0.00 (39)	0.09 ^b^
PR (ms)	70 ± 20 (70)	60 ± 10 (60)	60 ± 10 (60)	60 ± 0.00 (62)	0.06 ^b^
QRS (ms)	20 ± 20 (48)	40 ± 0.00 (40)	0.04 ± 0.00 (40)	40 ± 0.00 (41)	0.41 ^b^
QT (ms)	180 ±50 (184)	160 ± 25 (160)	150 ± 100 (150)	160 ± 20 (166)	0.02 ^b^*
P (mV)	0.10 ± 0.04 (0.09)	0.11 ± 0.03 (0.10)	0.09 ± 0.02 (0.10)	0.09 ± 0.03 (0.098)	0.96 ^b^
R (mV)	0.23 ± 0.18 (0.21)	0.29 ± 0.21 (0.23)	0.24 ± 0.17 (0.22)	0.26 ± 0.18 (0.219)	0.92 ^b^
T (mV)	0.19 ± 0.23 (0.094)	0.09 ± 0.05 (0.08)	0.06 ± 0.04 (0.06)	0.07 ± 0.06 (0.058)	0.00 ^b^*

Mean ± SD: standard deviation (median), (*) significant difference (*p* < 0.05), and Mann–Whitney (^b^).

**Table 6:** Electrocardiographic comparison of the CG versus the OG M0 cohort, in order of the mean, standard deviation, and median.

ECG/OG	CG (n = 25)	O GM0 (n = 40)	*p*
	Mean ± SD (Median)	Mean ± SD (Median)	
Axis (°)	66.48 ± 33.02 (60)	25.15 ± 76.94 (54)	0.11 ^b^
P (ms)	40 ± 5 (50)	45 ± 10 (40)	0.22 ^b^
PR (ms)	70 ± 12 (77)	70 ± 20 (70)	0.49 ^b^
QRS (ms)	40 ± 6 (60)	20 ± 20 (48)	0.00 ^b^*
QT (ms)	105 ± 34 (160)	108 ± 50 (184)	0.00 ^b^*
P (mV)	0.09 ± 0.029 (0.029)	0.10 ± 0.04 (0.09)	0.60 ^a^
R (mV)	0.29 ± 0.145 (0.276)	0.23 ± 0.18 (0.21)	0.05 ^b^
T (mV)	0.08 ± 0.051 (0.071)	0.19 ± 0.23 (0.094)	0.00 ^b^*

Mean ± SD: standard deviation (median), (*) significant difference (*p* < 0.05), *t* test (^a^), and Mann-Whitney test (^b^).

**Table 7:** Receiver operating characteristic (ROC) curves of biochemical parameters (urea, creatinine, and potassium) and cut-off points for determining the sinusoventricular rhythm with an atrial parade.

Parameter	Auroc (95% IC)	Best Cut-Off Point	Sensitivity (95% CI)	Specificity (95%) CI
Urea (mg/dL)	77% (63–92%)	277	66% (47–82%)	90% (55–99%)
Creatinine (mg/dL)	87% (75–99%)	11.91	73% (54–87%)	80% (44–97%)
Potassium (mmol/L)	87% (76–99%)	7.66	83% (65–94%)	70% (34–93%)

AUROC, area under the receiver characteristics curve; CI, confidence interval.

**Table 8:** Receiver operating characteristic (ROC) curves of biochemical parameters (urea, creatinine, and potassium) and cut-off points for the determination of right branch block volume.

Parameter	Auroc (95% IC)	Best Cut-Off Point	Sensitivity (95% CI)	Specificity (95%) CI
Urea (mg/dL)	85% (7–10%)	357	73% (54–87%)	60% (26–87%)
Creatinine (mg/dL)	62% (44–81%)	9.28	56% (37–74%)	90% (55–99%)
Potassium (mmol/L)	69% (41–98%)	5.95	56% (37–74%)	100% (69–100%)

**Table 9:** Accuracy of the biochemical parameters for LAFB determination.

Parameter	Auroc (95% IC)	Best Cut-Off Point	Sensitivity (95% CI)	Specificity (95%) CI
Urea (mg/dL)	71% (39–100%)	189	70% (50–85%)	0% (0–30%)
Creatinine (mg/dL)	71% (37–100%)	4.91	73% (54–87%)	0% (0–30%)
Potassium (mmol/L)	56% (12–100%)	5.75	56% (37–74%)	0% (0–30%)

AUROC, area under the receiver characteristics curve; CI, confidence interval.

**Table 10:** Comparison of HRV and SBP in cats in the OG at different times.

HRV/SBP Moments	M0 (n = 24) Mean ± SD (Median)	M1 (n = 34) Mean ± SD (Median)	M2 (n = 24) Mean ± SD (Median)	M3 (n = 15) Mean ± SD (Median)	*p*
HR (bpm)	199.13 ± 32.69 (198)	198 ± 29.22 (198)	210.23 ± 22.11 (207)	210.46 ± 21.12 (214)	0.30 ^b^
NN medium	309.68 ± 53.94 (303.5)	309.65 ± 48.03 (303.5)	288.14 ± 29.58 (290)	288.13 ± 33.04 (280)	0.30 ^b^
SDNN (ms)	7.09 ± 3.08 (6.05)	14.95 ± 12.65 (12.75)	11.18 ± 7.76 (9.1)	14.34 ± 8.94 (13.1)	0.03 ^b^*
rMSSD (ms)	6.26 ± 2.13 (5.7)	13.84 ± 14.08 (10.1)	9.31 ± 7.71 (7.4)	12.65 ± 9.29 (10)	0.03 ^b^*
PNN50%	0.15 ± 0.26 (0)	2.11 ± 4.39 (0.36)	0.92 ± 2.61 (0.24)	1.83 ± 3.30 (0.4)	0.17 ^b^
LF (nu)	51.49 ± 13.76 (51.22)	45.00 ± 15.04 (47.51)	49.83 ± 15.20 (52.68)	46.41 ± 14.61 (45.58)	0.09 ^b^
HF (nu)	48.43 ± 13.74 (48.75)	54.86 ± 14.99 (52.36)	47.40 ± 12.69 (46.49)	53.45 ± 14.47 (54.26)	0.09 ^b^
LF/HF	1.27 ± 0.86 (1.051)	0.96 ± 0.57 (0.907)	1.28 ± 0.72 (1.151)	1.02 ± 0.65 (0.84)	0.09 ^b^
SBP (mmHg)	120.82 ± 26.84 (120)	117.41 ± 24.43 (120)	127.36 ± 25.17 (121)	143.92 ± 19.82 (145)	0.03 ^b^*

Mean ± SD: standard deviation (median), (*) significant difference (*p* < 0.05); and Mann-Whitney test (^b^).

**Table 11:** Comparison of HRV and SBP in the CG and OG at M0.

HRV SBP	CG (n = 25) Mean ± SD (Median)	OG (n = 24) M0 Mean ± SD (Median)	*p*
HR (bpm)	188.6 ± 21.42 (187)	199.136 ± 32.691 (198)	0.19 ^a^
NN medium	322.2 ± 37.543 (320)	309.682 ± 53.949 (303.5)	0.35 ^a^
SDNN (ms)	11.396 ± 4.135 (9.7)	7.095 ± 3.081 (6.05)	0.00 ^b^*
rMSSD (ms)	9.88 ± 4.594 (9)	6.264 ± 2.138 (5.7)	0.00 ^b^*
PNN50%	0.472 ± 0.843 (0.26)	0.152± 0.265 (0)	0.05 ^b^
LF (nu)	53.75 ± 15.84 (57.55)	51.492 ± 13.766 (51.22)	0.60 ^a^
HF (nu)	46.16 ± 15.799 (42.42)	48.435 ± 13.742 (48.75)	0.60 ^a^
LF/HF	1.413 ± 0.817 (1.357)	1.274 ± 0.86 (1.051)	0.35 ^b^
SBP (mmHg)	132.12 ± 18.83 (130)	120.82 ± 26.847 (120)	0.03 ^b^*

Mean ± SD: standard deviation (median), (*) significant difference (*p* < 0.05), *t* test (^a^), and Mann-Whitney test (^b^).

## Data Availability

The original contributions presented in the study are included in the article, further inquiries can be directed to the corresponding author.
